# Supplementary material for: Prophylactic versus therapeutic dose anticoagulation effects on survival among critically ill patients with COVID-19
Source: PLoS One. 2022 Jan 19;17(1):e0262811. doi: 10.1371/journal.pone.0262811 (PMC8769345; doi:10.1371/journal.pone.0262811)
Supplement: Attachment — Submitted filename: Response reviewers - R1.docx [file pone.0262811.s001.docx]

July 27, 2021

To: Dr. Giuseppe Remuzzi, Academic Editor of *PLOS ONE*

Re: Manuscript revision **PONE-D-21-15647**

“Clinical Characteristics and Survival Analysis of Critically Ill COVID-19 Patients by Anticoagulant Dose”

Dear Dr. Giuseppe Remuzzi:

We appreciate the thoughtful comments by the reviewers and editorial office. We have carefully revised the manuscript to address any concerns and suggested edits. Detailed point-by-point responses are presented below. Changes in the revised manuscript are highlighted in red color.

There are no ethical or legal restrictions on sharing a de-identified data set. An anonymized data set necessary to replicate our study findings will be made available.

Sincerely,

Dr. Tim Duong

Dr. Wouter Hoogenboom

Joyce Lu

On behalf of all authors

**Academic Editor**

1. **Concern about the conclusions since this type of study cannot lend support or discourage the use of prophylactic versus therapeutic use of anticoagulants;**

**Response**: We agree that the observational nature of our study does not allow recommendations regarding anticoagulant treatment. Therefore, we modified the conclusions throughout the manuscript:

Abstract:

“Our findings raise the possibility that prolonged use of high dose AC, independent of thrombotic events or clinical background, might be associated with higher risk of in-hospital mortality.~~, and do not support routine and long-term use of therapeutic dose AC in COVID-19 patients admitted to the ICU~~.”

Discussion (first paragraph):

“This study described the clinical characteristics and survival probability by anticoagulant treatment of 311 critically ill patients with COVID-19 hospitalized at Stony Brook University Hospital between February 7, 2020, and June 30, 2020. . . ~~Our findings do not support routine long-term use of therapeutic dose AC in unselected patients with severe COVID-19.~~”

2. **Concern about the fact that the worse clinical background is causing both the poor survival and the selection of the therapeutic regimen;**

**Response**: In our cohort, only COPD was significantly more prevalent in the therapeutic group. We performed additional analysis with COPD and cumulative comorbidities (i.e., hypertension, diabetes, asthma, coronary artery disease, COPD, heart failure, cancer, immunosuppression, and chronic kidney disease) as additional covariates to control for clinical background. We found that including these covariates did not change the results. That is, the therapeutically anticoagulated cohort with 3 or more weeks of ICU admission had significantly higher risk of death compared to the prophylactically anticoagulated cohort in Cox proportional hazard model with age and comorbidities as covariates:

With age and COPD as covariates: adj. HR = 5.04, 95% CI = 1.75 to 14.51, p = 0.003

With age and cumulative comorbidities as covariates: adj. HR = 4.82, 95% CI = 1.66 to 13.89, p = 0.004.

It is likely that elevated D-dimer at admission contributed to the selection of therapeutic regimen, but D-dimer and age as covariates in Cox regression did not change the results (HR = 4.71, 95% CI = 1.61 to 13.77, p = 0.005), nor did D-dimer, comorbidities and age as covariates (HR = 5.22, 95% CI = 1.72 to 15.82, p = 0.003).

We included this additional analysis in the Results section on page 11 to support our findings:

“Therapeutic AC patients had nearly 5 times higher risk of death than prophylactic AC patients (adj. HR = 4.89, 95% CI = 1.71 to 14.0, p = 0.003) in Cox regression adjusted for age. This result remained unchanged after including additional covariates to control for clinical status, including thrombotic events (adj. HR = 4.18, 95% CI = 1.44 to 12.1, p = 0.009), and D-dimer and cumulative comorbidities (HR = 5.22, 95% CI = 1.72 to 15.82, p = 0.003).”

3. **Unclear how the equal proportion of therapeutic versus prophylactic regimens was occurring;**

**Response**: The approximately equal group proportions were not predetermined, but a coincidental result after applying study exclusion criteria and grouping patients by AC dose, as described in more detail in the ‘Study Population and data collection’ of the Methods section (page 6) and illustrated in Figure 1.

4. **Need to provide data about the dosage of the heparin drip, Eliquis, Xarelto, dabigatran;**

**Response**: Anticoagulant dosage varied in time and across patients. Dosage was continuous in some patients and changing in others. Patients were grouped as low dose (continuous low dosage only) or high dose (continuous high dose or change to high dose) based on the criteria described in the text. We revised text in the Methods section (page 5) to clarify patient grouping based on dosage:

“Patients were divided into one of two groups based on AC treatment dosage received: (1) continuous and exclusively low dose / prophylactic anticoagulation – that is, subcutaneous heparin or Lovenox (enoxaparin) at a dose of 40 mg twice daily; or (2) high dose / therapeutic anticoagulation – that is, 1 mg/kg twice daily or 1.5 mg/kg daily of the following anticoagulants: any heparin drip, Eliquis (apixaban), Xarelto (rivaroxaban), Pradaxa (dabigatran), Coumadin (warfarin), or Lovenox (enoxaparin)). Patients who received both prophylactic and therapeutic dosages during their hospital stay were grouped in the therapeutic cohort for this analysis.”

5. **Need to highlight more useful messages;**

**Response**: We agree and amended the text to clarify and highlight messages. Of note, due to the observational nature of this study, we can only report the existence of associations between exposure and outcomes:

Abstract (page 2, Results):

“For the first 3 weeks of ICU stay, we observed similar survival curves for prophylactic and therapeutic AC groups. However, after 3 or more weeks of ICU stay, the therapeutic AC group, characterized by high incidence of acute kidney injury, had markedly higher death incidence rates with 8.6 deaths (95% CI=6.2-11.9 deaths) per 1,000 person-days and about 5 times higher risk of death (adj. HR=4.89, 95% CI=1.71-14.0, p=0.003) than the prophylactic group (2.4 deaths [95% CI=0.9–6.3 deaths] per 1,000 person-days). Non-survivors were characterized by older males with depressed lymphocyte counts and cardiovascular disease.”

Abstract (page 2, Conclusions):

“Our findings raise the possibility that prolonged use of high dose AC, independent of thrombotic events or clinical background, might be associated with higher risk of in-hospital mortality. Moreover, AKI, age, lymphocyte count, and cardiovascular disease may represent important risk factors that could help identify at-risk patients who require long-term hospitalization with therapeutic dose AC treatment.”

Discussion (page 12):

“We made the following primary study observations: (1) Therapeutic dose AC regimens did not provide a survival benefit over prophylactic dose AC regimens in critically ill COVID-19 patients; (2) The survival curve of the therapeutic cohort diverged from the prophylactic cohort after 3 weeks indicating higher mortality after extended ICU care, which could not be explained by clinical background or thrombotic events alone; (3) Therapeutic dose patients had higher incidence of hospital-acquired AKI; and (4) Non-survivors in the therapeutic cohort with extended ICU care were characterized by older males with depressed lymphocyte count and cardiovascular disease, which represent risk factors that may help identify at-risk patients who require long-term hospitalization with therapeutic dose AC treatment.”

6. **Unclear the reasons for the higher mortality with therapeutic anticoagulation**

**Response**: We expanded the discussion on possible reasons for the higher mortality with therapeutic anticoagulation (page 14):

“There are different possible explanations for the higher mortality observed among therapeutically anticoagulated patients in our cohort. Compared to the prophylactic cohort, the therapeutic cohort was older, proportionally more male, and had higher prevalence of preexisting conditions, which are known risk factors for worse COVID-19 outcomes (Parohan *et al*., 2020). Therapeutic users also had higher incidence of hospital acquired AKI, which is known to be associated with increased risk of COVID-19 related mortality (Chan *et al*., 2021). Whether prolonged use of high dose AC is causally related with higher risk of in-hospital death, possibly mediated by acute kidney injury, requires further studies and randomized controlled trials.”

New references added:

Parohan M, Yaghoubi S, Seraji A, Javanbakht MH, Sarraf P, Djalali M. Risk factors for mortality in patients with Coronavirus disease 2019 (COVID-19) infection: a systematic review and meta-analysis of observational studies. Aging Male. 2020 Dec;23(5):1416-1424. doi: 10.1080/13685538.2020.1774748. Epub 2020 Jun 8. PMID: 32508193.

Chan L, Chaudhary K, Saha A, Chauhan K, Vaid A, Zhao S, Paranjpe I, Somani S, Richter F, Miotto R, Lala A, Kia A, Timsina P, Li L, Freeman R, Chen R, Narula J, Just AC, Horowitz C, Fayad Z, Cordon-Cardo C, Schadt E, Levin MA, Reich DL, Fuster V, Murphy B, He JC, Charney AW, Böttinger EP, Glicksberg BS, Coca SG, Nadkarni GN; Mount Sinai COVID Informatics Center (MSCIC). AKI in Hospitalized Patients with COVID-19. J Am Soc Nephrol. 2021 Jan;32(1):151-160. doi: 10.1681/ASN.2020050615. Epub 2020 Sep 3. PMID: 32883700; PMCID: PMC7894657.

7. **Concern about the fact that bleeding is not a significant cause of morbidity in hospitalized COVID-19 patients;**

**Response**: Patients with bleeding events did not receive anticoagulation or required a pause in anticoagulation during hospital stay and were excluded from analysis. Of the remaining patients, we are unable to provide data about other significant bleeding events because if present they were not significant enough to warrant discontinuation of anticoagulation. We updated the methods and discussion section to clarify the text:

Methods section (page 5)

“Patients with contraindications for anticoagulant treatment who did not receive anticoagulation or who required a pause in anticoagulation during hospital stay were excluded from the analysis.”

Discussion (page 14):

“While anticoagulants are effective in lowering rates of venous thromboembolism (VTE) [7], they may pose risk for bleeding, which could complicate hospitalization for COVID-19, although randomized trials are needed to confirm this [24].”

8. **Concern about the fact that anticoagulant-related nephropathy (ARN) is a known concept, but no studies have reported this complication in COVID-19;**

**Response**: We agree and modified the discussion (page 14) to read:

“Over-anticoagulation, linked to profuse glomerular hemorrhage and anticoagulant-related nephropathy (ARN)—a newly recognized form of acute kidney injury [25]—is another concern for hospitalized COVID-19 patients. However, no studies have reported this complication in COVID-19 and, despite high incidence of AKI in the therapeutic cohort, our data is inconclusive regarding ARN as a possible complication of COVID-19 among anticoagulated patients due to lack of data on over-anticoagulation. More studies are needed to explore the possibility of over-anticoagulation as a complication of COVID-19.”

9. **Need to provide data about over-anticoagulation;**

**Response**: The status of overanticoagulation could not be determined from available data in the patient charts. Please see previous point 8 for a modified discussion.

10. **Unclear whether there was no selection bias in the groups that received prophylactic versus therapeutic dose anticoagulation;**

**Response**: Dose regimens were based on general risk assessment considering clinical background, preexisting conditions and presenting laboratory values. Furthermore, potential confounder (e.g., age, d-dimer and comorbidities) were used as covariates in analysis and now described in the Results (page 10). We further acknowledge that unintentional patient selection bias is inherent to retrospective studies and suggested that randomized controlled trials are needed to assess causal relationships. We expanded the Methods and Limitations section to read:

Methods section (page 5):

“Dose regimens were based on general risk assessment considering clinical background, preexisting conditions, and presenting laboratory values.”

Limitations section (page 14):

“As with any retrospective study, there could be unintentional patient selection bias, and therefore, randomized controlled trials are needed to assess causal relationships between various AC treatment regimens and COVID-19-related survival and long-term outcomes. Also inherent to observational studies is the potential for unintentional confounding variables, such as group differences in age, although we attempted to control for this in covariate adjusted analysis.”

11. **Unclear whether older people and those with higher D-dimers were given therapeutic dose.**

**Response**: As indicated in point 10, dose regimens were based on general risk assessment considering clinical background, preexisting conditions, and presenting laboratory values. Furthermore, potential confounders such as age and d-dimer were used as covariates in analysis. We amended the Methods (page 5) to read:

“Dose regimens were based on general risk assessment considering clinical background, preexisting conditions, and presenting laboratory values.”

Journal requirements:

1. **Please ensure that your manuscript meets PLOS ONE's style requirements, including those for file naming.**

**Response**: We revisited the PLOS ONE style and submission guidelines and updated the manuscript accordingly.

2. **We note that you have indicated that data from this study are available upon request. PLOS only allows data to be available upon request if there are legal or ethical restrictions on sharing data publicly.**

**Response:** There are no ethical or legal restrictions on sharing a de-identified data set. We will make available an anonymized data set necessary to replicate our study findings.

**Reviewer 1**

**Major**

1. **Although the authors want to say that therapeutic anticoagulation is not ideal, I feel there are more useful messages in here which may be highlighted. For example, the survival curves diverge after three weeks with the two doses. Secondly, there were more kidney injury (not sure how it can be conclusively said to be due to ARN – see below)**

**Response**: We agree and amended the text to clarify and highlight messages. Of note, due to the observational nature of this study, we can only report the existence of associations between exposure and outcomes:

Abstract (page 2, Results):

“For the first 3 weeks of ICU stay, we observed similar survival curves for prophylactic and therapeutic AC groups. However, after 3 or more weeks of ICU stay, the therapeutic AC group, characterized by high incidence of acute kidney injury, had markedly higher death incidence rates with 8.6 deaths (95% CI=6.2-11.9 deaths) per 1,000 person-days and about 5 times higher risk of death (adj. HR=4.89, 95% CI=1.71-14.0, p=0.003) than the prophylactic group (2.4 deaths [95% CI=0.9–6.3 deaths] per 1,000 person-days). Non-survivors were characterized by older males with depressed lymphocyte counts and cardiovascular disease.”

Abstract (page 2, Conclusions):

“Our findings raise the possibility that prolonged use of high dose AC, independent of thrombotic events or clinical background, might be associated with higher risk of in-hospital mortality. Moreover, AKI, age, lymphocyte count, and cardiovascular disease may represent important risk factors that could help identify at-risk patients who require long-term hospitalization with therapeutic dose AC treatment.”

Discussion (page 12):

“We made the following primary observations: (1) Therapeutic dose AC regimens did not provide a survival benefit over prophylactic dose AC regimens in critically ill COVID-19 patients; (2) The survival curve of the therapeutic cohort diverged from the prophylactic cohort after 3 weeks indicating higher mortality after extended ICU care, which could not be explained by clinical background or thrombotic events alone; (3) Therapeutic dose patients had higher incidence of hospital-acquired AKI; and (4) Non-survivors in the therapeutic cohort with extended ICU care were characterized by older males with depressed lymphocyte count and cardiovascular disease, which represent risk factors that may help identify at-risk patients who require long-term hospitalization with therapeutic dose AC treatment.”

2. **Can the authors explain what may be the reasons for the higher mortality with therapeutic AC – although you state “investigated the cause of death and found no differences in cause of death between prophylactic and therapeutic groups”**

**Response**: We expanded the discussion on possible reasons for the higher mortality with therapeutic anticoagulation (page 14):

“There are different possible explanations for the higher mortality observed among therapeutically anticoagulated patients in our cohort. Compared to the prophylactic cohort, the therapeutic cohort was older, proportionally more male, and had higher prevalence of preexisting conditions, which are known risk factors for worse COVID-19 outcomes (Parohan *et al*., 2020). Therapeutic users also had higher incidence of hospital acquired AKI, which is known to be associated with increased risk of COVID-19 related mortality (Chan *et al*., 2021). Whether prolonged use of high dose AC is causally related with higher risk of in-hospital death, possibly mediated by acute kidney injury, requires further studies and randomized controlled trials.”

New references added:

Parohan M, Yaghoubi S, Seraji A, Javanbakht MH, Sarraf P, Djalali M. Risk factors for mortality in patients with Coronavirus disease 2019 (COVID-19) infection: a systematic review and meta-analysis of observational studies. Aging Male. 2020 Dec;23(5):1416-1424. doi: 10.1080/13685538.2020.1774748. Epub 2020 Jun 8. PMID: 32508193.

Chan L, Chaudhary K, Saha A, Chauhan K, Vaid A, Zhao S, Paranjpe I, Somani S, Richter F, Miotto R, Lala A, Kia A, Timsina P, Li L, Freeman R, Chen R, Narula J, Just AC, Horowitz C, Fayad Z, Cordon-Cardo C, Schadt E, Levin MA, Reich DL, Fuster V, Murphy B, He JC, Charney AW, Böttinger EP, Glicksberg BS, Coca SG, Nadkarni GN; Mount Sinai COVID Informatics Center (MSCIC). AKI in Hospitalized Patients with COVID-19. J Am Soc Nephrol. 2021 Jan;32(1):151-160. doi: 10.1681/ASN.2020050615. Epub 2020 Sep 3. PMID: 32883700; PMCID: PMC7894657.

**Minor**

**1. Avoid the term ‘coagulopathy’ which is vague**

**Response**: Thank you for this suggestion. We removed the term from the manuscript.

**2. Bleeding is not a significant cause of morbidity in hospitalized COVID-19 patients. Reference 24 does not support this**

**Response**: We revised the text to read:

“While anticoagulants are effective in lowering rates of venous thromboembolism (VTE) [7], they may pose risk for bleeding ~~and are a significant cause of morbidity in hospitalized COVID-19 patients~~, which could complicate hospitalization for COVID-19, although this needs to be confirmed in randomized trials [24].”

**3. “Over-anticoagulation, linked to profuse glomerular hemorrhage and anticoagulant-related nephropathy (ARN)—a newly recognized form of acute kidney injury [25]—is another concern for hospitalized COVID-19 patients.” – ARN is a known concept but no studies have reported this complication in COVID-19. If they have been, please quote**

**Response**: We agree and only speculated that over-anticoagulation could be a possible complication of COVID-19 that should be explored. We modified the discussion (page 14) to read:

“Over-anticoagulation, linked to profuse glomerular hemorrhage and anticoagulant-related nephropathy (ARN)—a newly recognized form of acute kidney injury [25]—is another concern for hospitalized COVID-19 patients. However, no studies have reported this complication in COVID-19 and, despite high incidence of AKI in the therapeutic cohort, our data is inconclusive regarding ARN as a possible complication of COVID-19 among anticoagulated patients due to lack of data on over-anticoagulation. More studies are needed to explore the possibility of over-anticoagulation as a complication of COVID-19.”

**4. Also, this paper has not shown any data about over-anticoagulation**

**Response**: The status of overanticoagulation could not be determined from available data in the patient charts. Please see previous point 3 for a modified discussion.

**5. It is interesting to note that almost equal numbers 158 and 153 received prophylactic and therapeutic dose AC. Please state there was no selection bias. As stated in the demographics, were the older people and those with higher D-dimers were given therapeutic dose?**

**Response**: The approximately equal group proportions were not predetermined, but a coincidental result after applying study exclusion criteria and grouping patients by AC dose, as described in more detail on page 6 and illustrated in Figure 1.

**Reviewer 2**

The manuscript entitled "Clinical Characteristics and Survival Analysis of Critically Ill COVID-19 Patients by Anticoagulant Dose" by Duong et al. describes the mortality after anticoagulant therapy in COVID-19 patients. The topic is of interest, and the results intriguing.

1. **My main critique of the manuscript pertains to the conclusions: this type of study cannot lend support or discourage the use of prophylactic vs. therapeutic use of anticoagulants. The authors can only report the association between the therapeutic AC regimen and the poor survival. The authors can only speculate why this association is present in the discussion section. Indeed, as the authors hypothesize, one possibility is that the therapeutic AC regimen is bad.**

**Response**: Thank you for pointing out this important aspect. We agree that the observational nature of our study does not allow recommendations regarding anticoagulant treatment. Therefore, we modified the conclusions throughout the manuscript:

Abstract:

“Our findings raise the possibility that prolonged use of high dose AC, independent of thrombotic events or clinical background, might be associated with higher risk of in-hospital mortality.~~, and do not support routine and long-term use of therapeutic dose AC in COVID-19 patients admitted to the ICU~~.”

Discussion (first paragraph):

“This study described the clinical characteristics and survival probability by anticoagulant treatment of 311 critically ill patients with COVID-19 hospitalized at Stony Brook University Hospital between February 7, 2020, and June 30, 2020. . . ~~Our findings do not support routine long-term use of therapeutic dose AC in unselected patients with severe COVID-19.~~”

2. **However, it is equally possible that patients treated at high dose AC (heparin drip, Eliquis / apixaban, Xarelto / rivaroxaban, dabigatran / Pradaxa, warfarin / coumadin, enoxaparin / Lovenox 1mg / kg twice or 1.5mg / kg) were also those with an initial bad clinical background. Indeed, these patients had greater D-dimer values and COPD. In this scenario, the worse clinical background is causing both the poor survival and the selection of the therapeutic regimen.**

**Response**: In our cohort, only COPD was significantly more prevalent in the therapeutic group. We performed additional analysis with COPD and cumulative comorbidities (i.e., hypertension, diabetes, asthma, coronary artery disease, COPD, heart failure, cancer, immunosuppression, and chronic kidney disease) as additional covariates to control for clinical background. We found that including these covariates did not change the results. That is, the therapeutically anticoagulated cohort with 3 or more weeks of ICU admission had significantly higher risk of death compared to the prophylactically anticoagulated cohort in Cox proportional hazard model with age and comorbidities as covariates:

With age and COPD as covariates: adj. HR = 5.04, 95% CI = 1.75 to 14.51, p = 0.003

With age and cumulative comorbidities as covariates: adj. HR = 4.82, 95% CI = 1.66 to 13.89, p = 0.004.

It is likely that elevated D-dimer at admission contributed to the selection of therapeutic regimen, but D-dimer and age as covariates in Cox regression did not change the results (HR = 4.71, 95% CI = 1.61 to 13.77, p = 0.005), nor did D-dimer, comorbidities and age as covariates (HR = 5.22, 95% CI = 1.72 to 15.82, p = 0.003).

We included this additional analysis in the Results section on page 11 to support our findings:

“Therapeutic AC patients had nearly 5 times higher risk of death than prophylactic AC patients (adj. HR = 4.89, 95% CI = 1.71 to 14.0, p = 0.003) in Cox regression adjusted for age. This result remained unchanged after including additional covariates to control for clinical status, including thrombotic events (adj. HR = 4.18, 95% CI = 1.44 to 12.1, p = 0.009), and D-dimer and cumulative comorbidities (HR = 5.22, 95% CI = 1.72 to 15.82, p = 0.003).”

3. **I also noticed that the number of patients treated with enoxaparin 40mg twice was very much comparable with that of enoxaparin 1 mg/kg twice. This is intriguing as doctors apparently selected the AC regimen at random (if we hypothesize that the two groups of patients have overlapping clinical characteristics). I would ask the authors to explain how this equal proportion of therapeutic vs. prophylactic regimens was occurring.**

**Response**: The approximately equal group proportions were not predetermined, but a coincidental result after applying study exclusion criteria and grouping patients by AC dose, as described in more detail in the ‘Study Population and data collection’ of the Methods section (page 6) and illustrated in Figure 1.

We added clarification on how dose regimens were determined by the treating clinicians. Methods section (page 5):

“Dose regimens were based on general risk assessment considering clinical background, preexisting conditions, and presenting laboratory values.”

We further acknowledge the existence of unintentional patient selection bias inherent to retrospective studies. Limitations section (page 14):

“As with any retrospective study, there could be unintentional patient selection bias, and therefore, randomized controlled trials are needed to assess causal relationships between various AC treatment regimens and COVID-19-related survival and long-term outcomes. Also inherent to observational studies is the potential for unintentional confounding variables, such as group differences in age, although we attempted to control for this in covariate adjusted analysis.”

**Minor points:**

4. **No data are reported about the dosage of the heparin drip, Eliquis, Xarelto, dabigatran: please describe this info better.**

**Response**: Anticoagulant dosage varied in time and across patients. Dosage was continuous in some patients and changing in others. Patients were grouped as low dose (continuous low dosage only) or high dose (continuous high dose or change to high dose) based on the criteria described in the text. We revised text in the Methods section (page 5) to clarify patient grouping based on dosage:

“Patients were divided into one of two groups based on AC treatment dosage received: (1) continuous and exclusively low dose / prophylactic anticoagulation – that is, subcutaneous heparin or Lovenox (enoxaparin) at a dose of 40 mg twice daily; or (2) high dose / therapeutic anticoagulation – that is, 1 mg/kg twice daily or 1.5 mg/kg daily of the following anticoagulants: any heparin drip, Eliquis (apixaban), Xarelto (rivaroxaban), Pradaxa (dabigatran), Coumadin (warfarin), or Lovenox (enoxaparin)). Patients who received both prophylactic and therapeutic dosages during their hospital stay were grouped in the therapeutic cohort for this analysis.”

5. **In the list of drugs, sometimes you use first the commercial and then the official drug name, and sometimes the other way round (e.g., Eliquis / apixaban, Xarelto / rivaroxaban, dabigatran / Pradaxa, warfarin/coumadin). Please be consistent.**

**Response**: Thank you for pointing this out. We revised the text to be consistent:

“Patients were divided into one of two groups based on AC treatment dosage received: (1) continuous and exclusively low dose / prophylactic anticoagulation – that is, subcutaneous heparin or Lovenox (enoxaparin) at a dose of 40 mg twice daily; or (2) high dose / therapeutic anticoagulation – that is, 1 mg/kg twice daily or 1.5 mg/kg daily of the following anticoagulants: any heparin drip, Eliquis (apixaban), Xarelto (rivaroxaban), Pradaxa (dabigatran), Coumadin (warfarin), or Lovenox (enoxaparin). Patients who received both prophylactic and therapeutic dosages during their hospital stay were grouped in the therapeutic cohort for this analysis.”
