# Supplementary material for: Prophylactic versus therapeutic dose anticoagulation effects on survival among critically ill patients with COVID-19
Source: PLoS One. 2022 Jan 19;17(1):e0262811. doi: 10.1371/journal.pone.0262811 (PMC8769345; doi:10.1371/journal.pone.0262811)
Supplement: Attachment — Submitted filename: Response to reviewers R2.docx [file pone.0262811.s002.docx]

**Comments to the Author**

**Reviewer #1**: **All the comments have been adequately addressed. All the important aspects have been taken into consideration. No further changes are needed**

**Reviewer #2**: **In the revised manuscript the authors agree that the observational design used was not sufficient to discourage the use of prophylactic AC because of potential selection bias of the treatment and control groups.**

1. **I still find it confusing stating that any heparin drip should be delivered at 1mg/kg (Pag 5) Similarly, are you sure whether coumadin, eliquis,xarelto can be administered at 1mg/kg twice a day (fig 5)? If not, please specify which dose you have used.**

**Response**: Thank you for pointing this out and we apologize for the confusion. We revisited the Stony Brook database registry for COVID-19 positive patients and associated data codebooks to verify anticoagulant information recorded, and made the following corrections on page 5:

“Patients were divided into one of two groups based on AC treatment dosage received: (1) continuous and exclusively low dose / prophylactic anticoagulation – that is, subcutaneous heparin or Lovenox (enoxaparin) at a dose of 40 mg twice daily; or (2) high dose / therapeutic anticoagulation – that is a) any heparin drip; b) Eliquis (apixaban), Xarelto (rivaroxaban), Pradaxa (dabigatran), or Coumadin (warfarin) at a typical therapeutic dose strength; or c) Lovenox (enoxaparin) at a dose of 1 mg/kg twice daily or 1.5 mg/kg daily.”

Of note, exact dosage regimens for Eliquis (apixaban), Xarelto (rivaroxaban), Pradaxa (dabigatran), and Coumadin (warfarin) were not recorded in the registry, only that they were given for therapeutic anticoagulation. For our study, it was sufficient to know whether someone received prophylactic or therapeutic anticoagulation, which was clearly recorded in the registry. Due to the retrospective nature of this study, additional data on dose of anticoagulation for each patient is not available, which we believe does not affect the results or conclusions of our study, but it is a study limitation that we acknowledge in the discussion (page 14):

“This is a retrospective study performed in a single hospital, and therefore would need to be replicated in collaboration with multiple institutions to achieve better generalizability. The Stony Brook data registry recorded whether COVID-19 patients received prophylactic or therapeutic anticoagulation, but additional data on exact dose for each patient was not available. Since we treated the primary exposure as a binary variable (i.e., prophylactic vs therapeutic anticoagulation), the lack of dosage data did not affect the analysis in this study. The results of the present study identify a time-dependent association between survival and anticoagulant treatment regimen among severe COVID-19 patients in the ICU, which might provide useful guidance for future prospective studies that can consider further drug subtype analysis, such as new oral anticoagulants (NOACs) vs coumadin vs heparin effects on COVID-19 outcomes. As with any retrospective study, there could be unintentional patient selection bias, and therefore, randomized controlled trials are needed to assess causal relationships between various AC treatment regimens and COVID-19-related survival and long-term outcomes. Also inherent to cohort studies is the potential for unintentional confounding variables, such as group differences in age, although we attempted to control for this in covariate adjusted analysis.”

2. **Furthermore, before collecting data from NOACs, coumadin, and heparin users as if they are the same therapy, you should consider that 1) coumadin might give a procoagulant effect initially: if this group was worse than the others, maybe this should be taken into consideration (without heparin bridge). The INR should be reported in this group and if it was lower than the therapeutic range these patients might even be considered controls**

**Response**: Thank you for your suggestions. While we agree that coumadin may have a temporary procoagulant effect when started without a heparin bridge, most patients that were on coumadin had pre-existing conditions such as Afib and were already taking prophylactic coumadin. Therefore, we believe that any procoagulant effects among coumadin users in our sample are limited. Of note, only 7 individuals (4.6%) in our study used coumadin at any time during hospitalization, and the small number of this group is unlikely to change the results of this study. Finally, we further note that stratified analysis by anticoagulation drug (i.e., NOACs vs coumadin vs heparin) is an interesting research venue, but beyond the focus of the current study.

3. **In the heparin group, the PTT should be reported, and you should verify it was in the therapeutic range (see above)**

**Response**: Lab values in this retrospective study were collected at admission. For this reason, we believe PTT values would not be representative as they were collected before AC treatment for most people. Due to the retrospective nature of this study, unfortunately, we cannot collect additional information, which is a study limitation inherent to the retrospective design of this study, which we acknowledge in the discussion. Of note, if a patient would show a PTT associated with subtherapeutic range, the AC regimen would be adjusted according to hospital protocol. Therefore, despite lack of PTT data, we believe that clinical management would ensure therapeutic range for heparin users. We made the following clarifications in the text:

To indicate the timing when lab values were collected (page 6):

“We collected COVID-19 outcome variables that included mortality rate, ICU duration, oxygen therapy, invasive mechanical ventilator (IMV) use, and incidence of acute kidney injury (AKI). We also collected demographics (i.e., age, gender, ethnicity and race), chronic comorbidities (i.e., smoking history, diabetes, hypertension, asthma, chronic obstructive pulmonary disease (COPD), coronary artery disease (CAD), heart failure, cancer, immune- suppression, chronic kidney disease (CKD)), vitals (i.e., heart rate (HR), diastolic blood pressure (DBP), systolic blood pressure (SBP), respiratory rate, pulse oxygen saturation (SpO_2_) and temperature), coagulation parameters (D-dimer, platelet count) and other laboratory tests (i.e., creatinine, procalcitonin, aspartate transaminase (AST), alanine aminotransferase (ALT), ferritin, lactate dehydrogenase (LDH), white blood cell count (WBC), C-reactive protein (CRP), lymphocytes count, brain natriuretic peptide (BNP), albumin, hematocrit, bicarbonate, creatinine), and blood gas variables (i.e., pO_2_, pCO_2_, and pH). All patient characteristics, symptoms, vitals, and laboratory values were collected at admission.”

And to highlight important future directions (page 14):

“This is a retrospective study performed in a single hospital, and therefore would need to be replicated in collaboration with multiple institutions to achieve better generalizability. The Stony Brook data registry recorded whether COVID-19 patients received prophylactic or therapeutic anticoagulation, but additional data on exact dose for each patient was not available. Since we treated the primary exposure as a binary variable (i.e., prophylactic vs therapeutic anticoagulation), the lack of dosage data did not affect the results or conclusions reported in this study. The results of the present study identify a time-dependent association between survival and anticoagulant treatment regimen among severe COVID-19 patients in the ICU, which might provide useful guidance for future prospective studies that can consider further drug subtype analysis, such as new oral anticoagulants (NOACs) vs coumadin vs heparin effects on COVID-19 outcomes. As with any retrospective study, there could be unintentional patient selection bias, and therefore, randomized controlled trials are needed to assess causal relationships between various AC treatment regimens and COVID-19-related survival and long-term outcomes. Also inherent to cohort studies is the potential for unintentional confounding variables, such as group differences in age, although we attempted to control for this in covariate adjusted analysis.”

4. **Indeed, in the therapeutic anticoagulation group, survival was linked to lower D-dimer, suggesting that some coagulation effect was present (Table 4).
   We have only a Table describing survival parameters in the therapeutic dose group. Why you do not report a similar Table for the group without therapeutic anticoagulation?**

**Response**: Thank you for providing us the opportunity to clarify this point. Table 4 represents a follow-up analysis based on our primary discovery that therapeutic dose users have increased risk of death after 3 or more weeks of ICU admission compared to prophylactic dose users, adjusted for age, clinical status, D-dimer and comorbidities. The goal of Table 4 is to better understand what factors were associated with poor survival in this particular group of interest. Reporting survival parameters for prophylactic users with prolonged ICU stay is of less interest in our opinion, because this group has favorable survival. We amended the text on page 11 to clarify this point:

“To better understand what factors were associated with poor survival among therapeutic anticoagulant users with 3 or more weeks of ICU admission, we conducted follow-up analysis comparing survivors (n=35) and non-survivors (n=32) in this group of interest (**Table 4**). Non-survivors were characterized by significantly older age (p=0.008), higher prevalence of coronary artery disease (p=0.009), lower lymphocyte count (p=0.007), and higher incidence of myocardial infarction (p=0.005). The majority (87%) of non-survivors were male (p=0.08).”

Thank you for your thoughtful comments.

Comments to the Editor. We made the following additional revisions:

1. We made the title more specific to better reflect the study conducted.
   a. Old title: Dose and duration effects of anticoagulant treatment among critically ill COVID-19 patients
   b. New title: Prophylactic versus therapeutic dose anticoagulation effects on survival among critically ill patients with COVID-19
2. Minor text clarifications in the abstract:

**Introduction** Although patients with severe COVID-19 are known to be at high risk of developing thrombotic events, the effects of anticoagulation (AC) dose and duration on in-hospital mortality in critically ill patients remain poorly understood and controversial. The goal of this study was to investigate survival of critically ill COVID-19 patients who received prophylactic or therapeutic dose AC and analyze the mortality rate with respect to detailed demographic and clinical characteristics.

**Materials and Methods** We conducted a retrospective, observational study of critically ill COVID-19 patients admitted to the ICU at Stony Brook University Hospital in New York who received either prophylactic (n=158) or therapeutic dose AC (n=153). Primary outcome was in-hospital death assessed by survival analysis and covariate-adjusted Cox proportional hazard model.

**Results** For the first 3 weeks of ICU stay, we observed similar survival curves for prophylactic and therapeutic AC groups. However, after 3 or more weeks of ICU stay, the therapeutic AC group, characterized by high incidence of acute kidney injury (AKI), had markedly higher death incidence rates with 8.6 deaths (95% CI=6.2-11.9 deaths) per 1,000 person-days and about 5 times higher risk of death (adj. HR=4.89, 95% CI=1.71-14.0, p=0.003) than the prophylactic group (2.4 deaths [95% CI=0.9–6.3 deaths] per 1,000 person-days). Among therapeutic AC users with prolonged ICU admission, non-survivors were characterized by older males with depressed lymphocyte counts and cardiovascular disease.

**Conclusions** Our findings raise the possibility that prolonged use of high dose AC, independent of thrombotic events or clinical background, might be associated with higher risk of in-hospital mortality. Moreover, AKI, age, lymphocyte count, and cardiovascular disease may represent important risk factors that could help identify at-risk patients who require long-term hospitalization with therapeutic dose AC treatment.
